# Tibial edema in osteomeniscal impingement: how can we contribute?

**DOI:** 10.1590/0100-3984.2023.56.3e3-en

**Published:** 2023

**Authors:** Carolina Freitas Lins, Marcello Henrique Nogueira-Barbosa

**Affiliations:** 1 Escola Bahiana de Medicina e Saúde Publica (EBMSP), Clínica Delfin Medicina Diagnóstica, Salvador, BA, Brazil. Email: carollins06@gmail.com.; 2 Department of Medical Imaging, Hematology, and Clinical Oncology, Ribeirão Preto Medical School, University of São Paulo (USP), Ribeirão Preto, SP, Brazil. Email: marcello@fmrp.usp.br.

Bone marrow edema occurs due to an increase in fluid in the extracellular spaces, secondary to capillary permeability, and can result from various physiological or pathological conditions^(1)^. Among radiological imaging methods, magnetic resonance imaging (MRI) is considered the modality of choice for the detection of bone marrow edema, which is characterized by intermediate or low signal intensity on T1-weighted sequences and high signal intensity on fluid-sensitive MRI sequences, unlike hematopoietic marrow, which is characterized by intermediate signal intensity on fluid-sensitive sequences^(2)^. The interpretation of MRI scans might help identify the etiology of bone marrow edema, thus allowing orthopedists to adopt the appropriate measures^(3)^.

Osteomeniscal impingement occurs when a flap or meniscal fragment is displaced into the meniscotibial recess, promoting reactive, potentially reversible bone marrow edema in the adjacent tibia^(4)^. Clinically, the most common complaint is medial pain when flexing the knee with valgus stress^(4,5)^. It is important to recognize this diagnosis, because meniscal injuries with osteomeniscal impingement might respond well to arthroscopic debridement of the displaced meniscal fragment^(4,6)^. However, for an orthopedist to identify the meniscal flap during the arthroscopic procedure, it is fundamental that its MRI findings have been well described by a radiologist^(5,7)^.

The differential diagnosis of bone marrow edema is quite broad, even when it is caused by osteomeniscal impingement, in which case it might be confused with subchondral insufficiency fracture^(5,8)^. It is therefore necessary to develop criteria to allow that distinction to be made on the basis of the MRI aspects of tibial edema, because osteomeniscal impingement benefits from a surgical approach, whereas subchondral insufficiency fractures do not^(5)^.

The article “Where is tibial edema located in cases of osteomeniscal impingement?”, authored by Helito et al.^(9)^ and published in the current issue of Radiologia Brasileira, describes a retrospective analysis of the MRI scans of 40 patients who underwent knee arthroscopy because a meniscal fragment was displaced into the meniscotibial recess, promoting peripheral edema in the adjacent tibia, as quantified in the coronal and axial planes. In their article, the authors propose some qualitative and quantitative criteria for bone marrow edema due to osteomeniscal impingement: it always starts at the periphery of the meniscus; in the coronal plane, it is more extensive in the craniocaudal direction than in the mediolateral direction; and in the axial plane, it is most often located in the center of the posterior region of the medial tibial plateau.

The conclusions drawn by Helito et al.^(9)^ serve to alert radiologists to two essential aspects in the evaluation of knee MRI in the context of tibial subchondral bone marrow edema. The first aspect is the location and extent of the edema, which can differentiate osteomeniscal impingement from subchondral insufficiency fracture. The second aspect is the difficulty of confirming the presence of a meniscal fragment in the meniscotibial recess in some cases, such as those in which it mimics meniscal extrusion. That differentiation is fundamental because it can help define the orthopedic approach (as conservative or surgical).

The main limitation of the Helito et al.^(9)^ study is the relatively small number of patients involved. However, the measurements made by the two evaluators, as well as the results of the interobserver and intraobserver analyses, demonstrated the consistency of the positive findings presented. Other limitations include the absence of functional scales and the lack of postoperative imaging examinations to correlate with the extent of edema over time, although those limitations do not invalidate the results obtained. Future studies on this topic could compare patients with osteomeniscal impingement and those with subchondral insufficiency fracture, in terms of the location and extent of medial tibial edema, in order to characterize the two conditions and differentiate between them with greater precision.
